# Transcriptional engineering for value enhancement of oilseed crops: a forward perspective

**DOI:** 10.3389/fgeed.2024.1488024

**Published:** 2025-01-07

**Authors:** Charli Kaushal, Mahak Sachdev, Mansi Parekh, Harini Gowrishankar, Mukesh Jain, Subramanian Sankaranarayanan, Bhuvan Pathak

**Affiliations:** ^1^ Department of Biological Sciences and Engineering, Indian Institute of Technology Gandhinagar, Palaj, Gujarat, India; ^2^ Biological and Life Sciences Division, School of Arts and Sciences, Ahmedabad University, Ahmedabad, Gujarat, India; ^3^ Department of Plant Pathology, University of Florida, Gainesville, FL, United States

**Keywords:** transcription factors, genome editing, CRISPR/Cas9, oilseed crops, fatty acid biosynthesis, seed maturation, omics

## Abstract

Plant-derived oils provide 20%–35% of dietary calories and are a primary source of essential omega-6 (linoleic) and omega-3 (α-linolenic) fatty acids. While traditional breeding has significantly increased yields in key oilseed crops like soybean, sunflower, canola, peanut, and cottonseed, overall gains have plateaued over the past few decades. Oilseed crops also experience substantial yield losses in both prime and marginal agricultural areas due to biotic and abiotic stresses and shifting agro-climates. Recent genomic, transcriptomic, and metabolomics research has expanded our understanding of the genetic and physiological control of fatty acid biosynthesis and composition. Many oilseed species have inherent stress-combating mechanisms, including transcription factor regulation. Advances in genome editing tools like CRISPR/Cas9 offer precise genetic modifications, targeting transcription factors and binding sites to enhance desirable traits, such as the nutritional profile and chemical composition of fatty acids. This review explores the application of genome editing in oilseed improvement, covering recent progress, challenges, and future potential to boost yield and oil content. These advancements could play a transformative role in developing resilient, nutritious crop varieties essential for sustainable food security in a changing climate.

## 1 Introduction

Oil seeds account for more than 207.8 M Ha of the world’s cultivated area and production is estimated to be 666.7 million tons (FAO, 2023/24). Soybean is the major oil crop with a production of 425.4 million tons, followed by rapeseed at 87.2 million tons, sunflower seed at 50.4 million tons, cottonseed at 44.6 million tons, and groundnut at 50.3 million tons (USDA, 2023/24).

The oil crops accumulate the high-energy triacylglycerols (TAGs) consisting of three glycerol-bound esterified fatty acids (FAs) as the primary source of stored carbon. Plant-derived oils typically account for 20%–35% of dietary calorie intake and play a critical role in human nutrition. While the human body can synthesize omega-9 FA (oleic acid), it relies on dietary intake for essential omega-6 (linoleic) and omega-3 (α-linolenic) FAs (Saini and Keum, 2018). The unique omega-3, -6 and -9 FAs profiles of the plant-derived oils are linked to improved cardiovascular health, reduced cancer risk, and decreased inflammation (Tian et al., 2023). Seeds of various oilseed crops such as peanut (*Arachis hypogaea*), canola or rapeseed (*Brassica napus*), Ethiopian mustard (*Brassica carinata*), Chinese mustard (*Brassica juncea*)*,* false flax (*Camelina sativa*), soybean (*Glycine max*), sunflower (*Helianthus annus*) and cottonseed (*Gossypium hirsutum*) are rich in ‘common’ FAs, including palmitic acid (C16:0), stearic acid (C18:0), oleic acid (C18:1Δ^9^), linoleic acid (C18:2Δ^9,12^), and α-linolenic acid (C18:3Δ^9,12,15^) (Attia et al., 2021). Seed oils also contain structurally diverse FAs with unusual features, such as atypical carbon chain lengths (fewer than 16 or more than 18 carbons), varying degrees of unsaturation, double-bond positions, cis/trans orientations, acetylenic bonds, or unique side-chain decorations (e.g., epoxy, hydroxy, cyclopropene, and furan rings). These ‘unusual’ FAs hold high value as nutraceuticals, biofuels, and industrial chemicals (Cahoon and Li-Beisson, 2020).

Achieving environmentally sustainable food security for the growing world population needs pragmatic emphases on improving crop yields and nutritional quality. Changing climate conditions continue to exacerbate the effects of temperature, drought and salinity stress across marginal agricultural landscapes. The abiotic stress conditions also increase the crops’ susceptibility to existing pathogens and have been implicated in increased virulence of previously mild strains (Wu et al., 2020). These biotic and abiotic stresses significantly impact agricultural productivity, especially in staple oilseed crops like soybean, sunflower, canola, peanut, and cottonseed, causing substantial yield losses. Despite notable gains in yield through traditional breeding, oilseed crop productivity has plateaued in recent decades. Both prime and marginal agricultural areas experience considerable yield losses in oilseeds due to combined stressors, amplified by climate change. For example, season-long high temperatures of 32°C significantly reduce biomass and yield in peanuts (Prasad et al., 2003), while yield models suggest a 3.1% reduction in soybean yield for every 1°C increase in average temperature (Zhao et al., 2017).

Recent advances in genomic, transcriptomic, and metabolomics research have unveiled a wealth of information about the genetic and physiological regulation of FA biosynthesis in oilseed crops. Large-scale genome sequencing of key crops such as canola, groundnuts, soybean, flax, and sunflower offers valuable data on traits governing yield, oil composition, and stress resilience. This genomic information significantly enhances our ability to develop crop varieties better suited to withstand environmental stresses and improve productivity (Varshney et al., 2021; Bohra et al., 2022).

Traditionally, studies on lipid metabolism in oilseeds have focused on enhancing FA biosynthesis by either overexpressing or disabling specific genes (Ruiz-Lopez et al., 2014). Transgenic approaches for improving traits in oilseed crops face challenges like low consumer acceptance, stringent regulation protocols, and the labor-intensive process of integrating transgenes into elite varieties (Meesapyodsuk et al., 2018; Na et al., 2018; Shah et al., 2018; Kim et al., 2019). Clustered Regularly Interspaced Short Palindromic Repeats (CRISPR) and CRISPR-associated protein (Cas) provide a molecular toolkit for precise editing of genetic information (Mohanta et al., 2017). CRISPR/Cas-mediated editing approaches offer targeted bi-allelic/homozygous mutations in multiple alleles. By targeting transcription factor (TF) binding sites, CRISPR/Cas toolkits enable redesign of the desired traits, such as the nutritional composition of FAs. Additionally, editing pathogen effector binding sites can suppress disease susceptibility genes, boosting disease resistance and yield (Greenwood et al., 2023). Nuanced variations of CRISPR/Cas, such as CRISPR/Cas-mediated transcriptional activation (CRISPRa) (Selma et al., 2019; Pan et al., 2021; Xiong et al., 2021; Ren et al., 2022) and CRISPR-interference (CRISPRi) (Tang et al., 2017; Ming et al., 2020), hold immense potential in generating crop varieties that are disease resistant, stress-tolerant and nutritionally balanced, particularly benefiting developing regions.

Complex allopolyploid genomes of oilseed crops, analyzed through large transcriptomic datasets and machine learning models, can guide CRISPR/Cas-mediated high-throughput workflows and reveal post-editing effects for optimized phenotyping (O’brien et al., 2021). This review explores genome editing’s role in tackling current challenges and advancing oilseed crop improvement. By synthesizing progress, highlighting challenges, and proposing future directions, it offers insights into developing resilient, nutritious oilseed varieties essential for sustainable food security in a changing climate.

## 2 Fatty acid biosynthesis pathways

Oilseed crops contain over 200 FAs, with seeds storing up to 60% of their dry weight as TAGs during maturation (Ohlrogge and Jaworski, 1997; Kazaz et al., 2022; Zhou et al., 2023). FAs are classified as saturated (no double bonds) or unsaturated (one or more double bonds, e.g., Δ12,15). Genes encoding FA biosynthesis proteins are identified and functionally annotated across several plant species. Despite varied oil compositions, the processes of production, modification, TAG assembly, and FA storage are generally conserved in oilseed crops (Zhou et al., 2023). FA biosynthesis occurs in plastids and mitochondria, involving enzymes like acetyl-CoA carboxylase (ACCase) and fatty acid synthase (FAS) (Ohlrogge et al., 1979).

**FIGURE 1:**
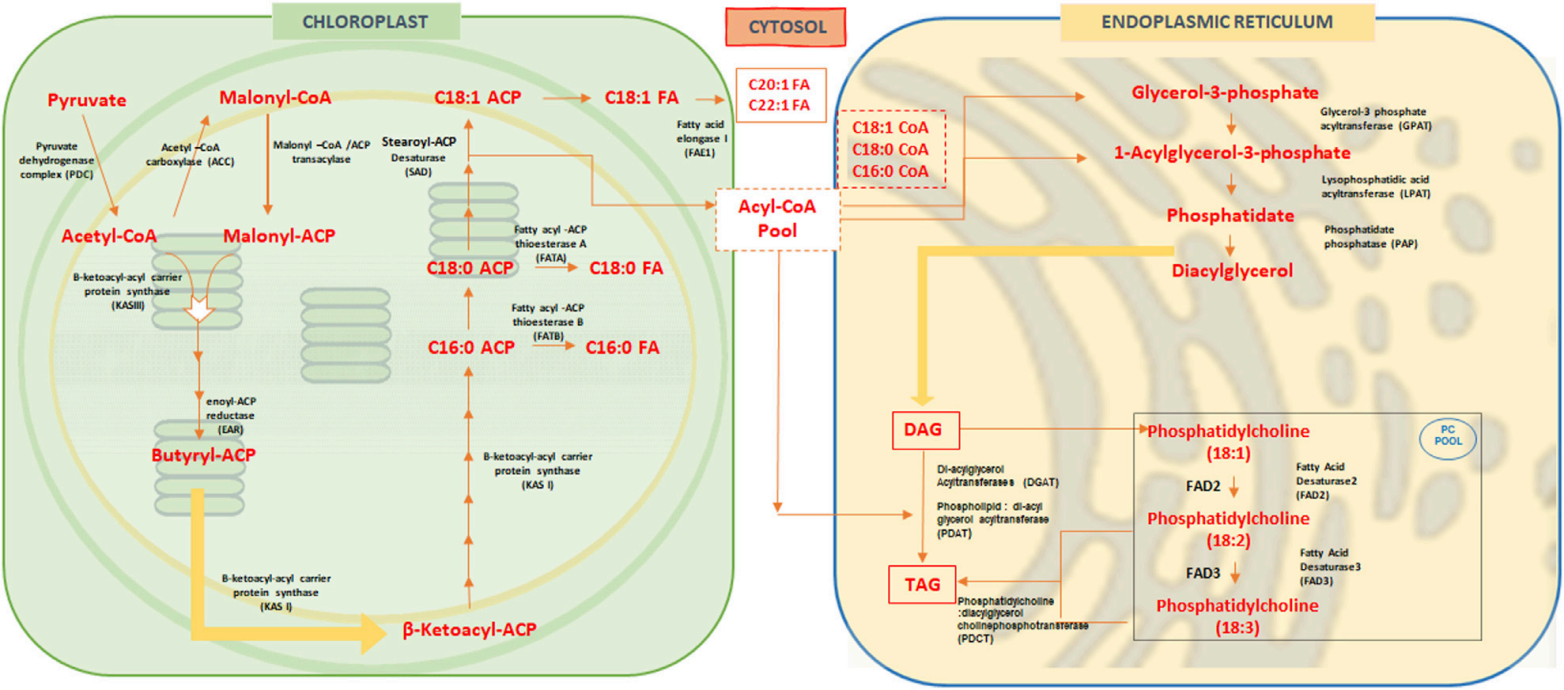
Overview of Fatty acid biosynthesis pathway in oilseed crops. In oilseed or higher plants, fatty acid biosynthesis occurs in the plastids, while polyunsaturated fatty acids (PUFAs) are synthesized in the endoplasmic reticulum (ER). In the chloroplasts, acetyl-CoA is first converted to malonyl-CoA by acetyl-CoA carboxylase. Malonyl-CoA is then converted to malonyl-ACP (acyl carrier protein), and two carbons are added after each cycle to the malonyl-acyl chain, synthesizing β-ketoacyl-ACP. This is followed by the formation of palmitic acid (C16:0), which is then elongated to stearic acid (C18:0) and subsequently desaturated to oleic acid (C18:1) by stearoyl-ACP desaturase (SAD). These fatty acids are transported to the cytosol, where further elongation occurs, converting them to C20:1 and C22:1 fatty acids by fatty acid elongase 1 (FAE1). Some fatty acids, such as C18:1-CoA, C18:0-CoA, and C16:0-CoA, are transported to the ER for desaturation. The acyl chains derived from these fatty acids are attached to glycerol-3-phosphate to form glycolipids. Through the action of enzymes like glycerol-3-phosphate acyltransferase (GPAT) and lysophosphatidic acid acyltransferase (LPAT), diacylglycerols (DAGs) are synthesized. The acyl chain C18:1 is then transferred to phosphatidylcholine (PC) and desaturated at the Δ-12 position to form C18:2 (linoleic acid) by FAD2 (fatty acid desaturase 2). This is further desaturated at the Δ-15 position to produce C18:3 (alpha-linolenic acid) by FAD3 (fatty acid desaturase 3). These acyl chains on phosphatidylcholine are subsequently used for the synthesis of triacylglycerols (TAGs).

ACCase catalyzes the ATP-dependent carboxylation of acetyl-CoA to malonyl-CoA. Acyl-carrier protein (ACP), an essential co-factor, accepts the malonyl group to form malonyl-ACP, which provides the thiol group for FA synthesis, forming thioesters with the elongating acyl chain (Lessire and Stumpe, 1983; Clough et al., 1992; Sasaki and Nagano, 2004; Park and Kim, 2022). Through a series of enzymatic steps, ACP-bound β-ketoacyl intermediates elongate by two-carbon units until the desired acyl length is achieved, aided by ketoacyl synthase (KS), reductase (KR), dehydratase (DH), and enoyl reductase (ER) enzymes (Harwood, 1996; Park and Kim, 2022). Finally, thioesterase (TE) terminates synthesis, producing free FAs like palmitic and stearic acids for export to the cytoplasm (Jones et al., 1995; Salas and Ohlrogge, 2002; Park and Kim, 2022) (Figure 1).

Stearic acid is desaturated in plastids to oleic acid (18:1Δ9) by stearoyl-ACP desaturase (SAD). Free FAs exit plastids via ABC transporter proteins (Jouhet et al., 2007) and are activated into acyl-CoAs by acyl-CoA synthetases for cytosolic processes (Schnurr et al., 2002). In the cytosol, acyl-CoAs bind to acyl-CoA-binding proteins (ACBPs), increasing solubility for transport, desaturation, and elongation in the endoplasmic reticulum (ER) (Fox et al., 2000; Bates et al., 2013; Vanhercke et al., 2019). Fatty acid export (FAX) family proteins in the ER, such as FAX2 and FAX4, aid FA transport during seed oil accumulation (Li et al., 2020), with desaturase enzymes like FAD2 and FAD3 converting oleic acid to linoleic and alpha-linolenic acids, respectively (Lemieux et al., 1990; Dar et al., 2017; Park and Kim, 2022).

Plant TAG biosynthesis from FAs occurs via both acyl-CoA-dependent and independent pathways. TAG biosynthesis intersects with membrane lipid production and affects the oil composition in seeds (Bates and Browse, 2012; Bates et al., 2013; Bates, 2016; Bates, 2022; Parchuri et al., 2024; Zhou et al., 2023). Acyltransferase enzymes in the ER transfer acyl-CoAs to glycerol-3-phosphate (G3P) to form TAG (Li-Beisson et al., 2013; Park and Kim, 2022). Glycerol-3-phosphate acyltransferase (GPAT) adds acyl-CoA to the G3P at the sn-1 position to produce lysophosphatidic acid (LPA) (Shockey et al., 2016; Park and Kim, 2022). Lysophosphatidic acid acyltransferase (LPAT) then transfers to acyl-CoA to the sn-2 position of LPA to form phosphatidic acid (PA). Phosphate phosphatase (PAP) dephosphorylates PA to produce diacylglycerol (DAG) (Carman and Han, 2006; Park and Kim, 2022). Lastly, diacylglycerol acyltransferase (DGAT) adds acyl-CoA to the sn-3 position of DAG to create TAG (Zou et al., 1999; Park and Kim, 2022) (Figure 1).

Empirical evidence suggests that phosphatidylcholine (PC) provides polyunsaturated fatty acids (PUFAs) in TAGs (Park and Kim, 2022). Lysophosphatidylcholine acyltransferases (LPCAT) catalysis releases PUFAs from PC thereby augmenting the acyl-CoA pool (Lager et al., 2013). In the ER, TAG is synthesized using acyl-CoA as an acyl donor and DAG as an acceptor, catalyzed by the enzyme acyl-CoA:diacylglycerol acyltransferase (Zou et al., 1999; Kim et al., 2005; Shockey et al., 2016; Park and Kim, 2022). Nonetheless, an acyl-CoA-independent mechanism for TAG biosynthesis has also been described in plants and in the yeast (*Saccharomyces cerevisiae*). Phospholipid:diacylglycerol acyltransferase (PDAT) converts PUFAs in PC to DAG, directly producing TAG (Dahlqvist et al., 2000; Park and Kim, 2022).

## 3 Omics-driven insights into fatty acid biosynthesis in oilseed crops

Recent advances in genomics, transcriptomics, proteomics, and metabolomics, have provided significant insights into the regulatory networks involved in fatty acid biosynthesis during oilseed maturation and enrichment (Liu N. et al., 2022; Turquetti-Moraes et al., 2022; Bu et al., 2023; Li et al., 2024). Through these omics approaches, understanding of oilseed crop genomes provide comparative insights into the evolution of genetic mechanisms controlling lipid biosynthesis, accumulation, and species-specific variations in lipid profiles. Constructing gene regulation networks can help identify highly connected gene hubs related to oil biosynthesis (Zuo et al., 2024).

In peanut (*Arachis hypogaea* L.), one of the world’s major oilseed crops, salt stress poses a significant challenge to growth, particularly in regions prone to saline soils. Transcriptome analysis of peanut seedlings under salt stress (250 mM NaCl for 4 days, S4) and subsequent recovery (3 days post-transfer to standard conditions, R3) revealed 1,742 differentially expressed genes (DEGs) under stress and 390 during recovery (Sui et al., 2018). Among these, genes encoding ω-3 fatty acid desaturase, crucial for converting linoleic acid (18:2) to alpha-linolenic acid (18:3), were downregulated under salt stress and upregulated during recovery, shedding light on the genetic basis of salt tolerance and fatty acid biosynthesis in peanuts.

In other oilseed crops, such as *Camellia oleifera* and olives, integrative omics approaches have elucidated the molecular mechanisms of fatty acid accumulation and oil quality. A combination of transcriptome sequencing (RNA-seq) and isobaric tags for relative and absolute quantification (iTRAQ) during seed maturation revealed a reduction in flavonoids and a corresponding increase in fatty acid accumulation (Ye et al., 2021). A meta-analysis of RNA-seq data identified key genes associated with oil quality in olives by comparing three growth stages (S1, S2, and S3). The analysis revealed 155, 473, and 241 DEGs across these comparisons, which were classified into four groups based on their roles in oil biosynthesis and quality determinants. Pathways like galactose metabolism and FA biosynthesis were highlighted, showcasing the olive’s efficiency in FA production (Asadi et al., 2023). Transcriptome and proteome analyses in peony seeds have identified key genes involved in unsaturated FA biosynthesis, with *FAD* genes playing a crucial role in alpha-linolenic acid (ALA) accumulation. An integrated analysis of transcriptomic and proteomic data identified 38,482 unigenes and 2,841 proteins across nine developmental stages. These were grouped into three developmental clusters, revealing 211 genes and 35 proteins linked to fatty acid metabolism, with a particular emphasis on genes involved in unsaturated fatty acid (UFA) and ALA biosynthesis. Notably, eight *FAD* genes exhibited peak expression 53 days after pollination, indicating their crucial role in ALA accumulation (Wang X. et al., 2019; Wang et al., 2019). Similarly, in peanuts, iTRAQ proteomics identified fatty acid biosynthesis 2 (FAB2) as a critical enzyme in oleic acid biosynthesis during early seed development in high-oleate cultivars (Liu et al., 2018).

Metabolomic analyses across different stages of seed development and maturation, combined with transcriptomic profiles, have deepened our understanding of lipid biosynthesis. By integrating transcriptome and metabolome profiles at different stages of seed maturation, a comprehensive understanding of the molecular mechanisms underlying lipid biosynthesis can be achieved. Such studies have shown that changes in metabolomes correspond to gene expression associated with lipid biosynthesis, aiding in the identification of TFs involved in these processes. This approach can identify critical time intervals for gene engineering-based breeding strategies (Tan et al., 2019; Jia C. et al., 2024). Thus, integrating genomics, transcriptomics, proteomics, and metabolomics data offers a holistic view of the molecular mechanisms that govern seed oil quality.

## 4 Transcriptional and epigenetic regulation of seed oil biosynthesis

### 4.1 Transcriptional regulation of fatty acid biosynthesis

**FIGURE 2:**
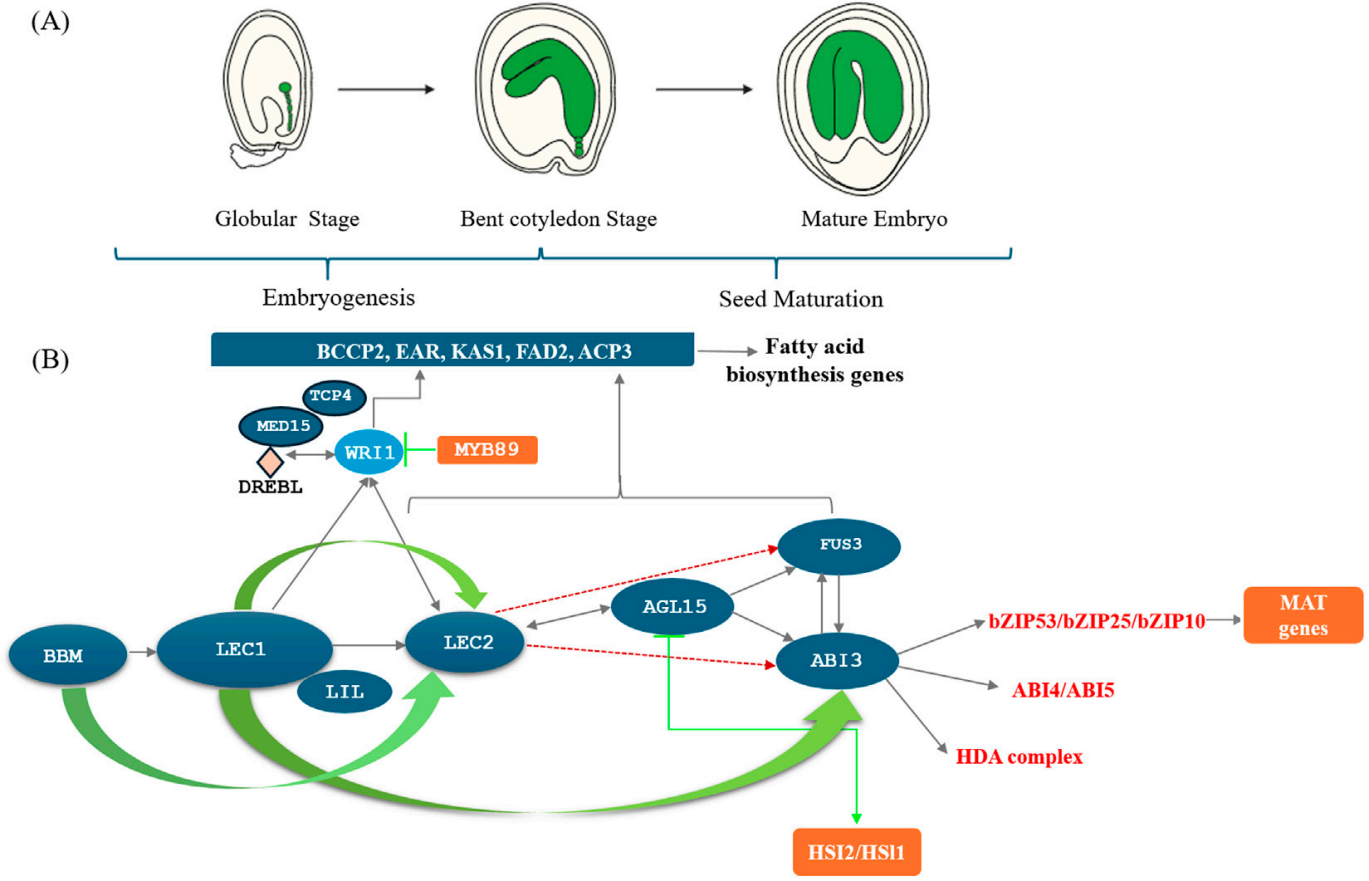
Networking of Transcription factors during oilseed maturation and Transcriptional regulation of seed development in *Arabidopsis thaliana.* **(A)** Stages of seed development in *Arabidopsis thaliana*: This figure shows broad stages of embryo development in *Arabidopsis.* Stage1: Globular stage, Stage 2: Bent cotyledon stage, Stage 3: Mature embryo. **(B)** Transcription Factor Network during Oilseed Maturation: Seed development is broadly categorized into two stages in seeds of *A. thaliana* and other crop plants. Stage 1- Embryogenesis, Stage 2-Seed maturation involving globular stage, bent cotyledon stage and mature embryo. Seed specific transcription factors include B3-domain TF (B3-domain containing transcription factors); LEC (Leafy cotyledons) - *LEC1*, *LEC2* and *LIL/FUS3*; *NF-Y* TF (Nuclear factor–Y)*-NF-YB/NF-YC*; *AP2/ERF* (APETALA2/ETHYLENE RESPONSE FACTOR) –*WRI1/BBM* (Baby Boom TF), *ARF*(Auxin Response Factors),*WRKY* TF, *MYB* TF (myeloblastosis TF); *bZIP* TF (Basic leucine zipper transcription factor), ABA (Abscisic acid insensitive) -*ABI3/4/5*; *AGL* TF (Agamous-Like TF) -*AGL15/18* play important role during different stages of seed development. *LEC1* and *BBM* (AP2-ERF TF) initiate the embryo formation or embryogenesis followed by coordinated expression of vital transcription factors like *LAFL-LEC2*, *ABI3*, *FUS3* and *LIL/LEC1* network initiating embryo maturation. The BABY BOOM (*BBM*) transcription factor regulates embryo formation and seed development and is required for embryo initiation and maintenance. The figure is created with bioRender.com.

Oilseeds are important sources of seed-specific proteins, TAGs, and other energy reserves that accumulate during embryogenesis. Fatty acid biosynthesis is a complex, multi-level process regulated at the transcriptional level by various transcription factors (Table 1). The seed maturation and embryogenesis processes are also regulated by a network of transcription factors (Verma et al., 2022). Key B3 domain-containing transcription factors, such as *ABI3*, *FUS3*, and *LEC2*, are essential in regulating seed maturation, transitioning from cell division to storage accumulation (Figure 2A). In dicotyledonous plants, seed development involves two main phases: morphogenesis, with cell division and expansion, followed by seed maturation, marked by storage component accumulation and preparation for desiccation tolerance (Pathak et al., 2020).

**TABLE 1:** Transcription factors and Epigenetic regulators of seed fatty acid accumulation.

S. No	Transcription factors	Family	Direct targets	Direct binding site	Role	References
(A)	Positive TF’s					
1	*ABI3* *Abscisic acid Insensitive 3*	B3-Domain transcription factor family protein	*ABI5, WRI1, FUS3, Cytochrome p450, FAD3, MYB30, LEA, CSD2, MYB73, Oleo2, DOG1*	Long RY motif-CATGCATG Short RY motif-CATGCA Less conserved RY motif- core CATGCA G-Box-ACGT	ABA perception and response Seed development, Seed oil biogenesis embryo development, Seed dormancy lipid Storage	Sasnauskas et al. (2018), Tian et al. (2020)
2	*FUS3*	B3 Domain transcription factor family protein	*VAL1, AGl15, LBD40, LEC1, LIL, EEL* *BBM, WRI1, ABI3*	RY motifs having a CATGCA(TG) sequence G-Box- ACGT	Post embryo development, Abiotic stress, Seed development, embryo development, lipid localization	Yamamoto et al. (2010), Wang and Perry (2013)
3	*LEC1*	Nuclear Transcription factor (NF-Y) family Subunit of Nuclear transcription factor NF-YB(HAP3)	*ABI3, bZIP67, EEL, FUS3*	CCAAT-box G-Box RY-motif	Photosynthesis, Chloroplast biogenesis during early seed development	Pelletier et al. (2017)
4	*LEC2*	B3 Domain transcription factor family protein	*WRI1, AT2S1, AT2S2, AT2S3, At2S4 AGL15, EEL, LOB40, IAA30, Oleo2*	RY-motif 1 kb upstream of TSS	Somatic embryo formation, embryo maturation	Kumar et al. (2020)
5	*WRI1*	APETALA2/ethylene-responsive element-binding protein (AP2/EREBP) family	*PKP1, bZIP67, GPDH, BCA5, ROD1, PKpPK-α, LEC1, LIL, TA2*	ASML1/WRI1 (AW)-box [5′-CNTNG(N)₇CG-3′, N = A, T, C or G] (−1 to −500bp upstream)	Enzymes involved in conversion of sugars to fatty acid. Fatty acid biosynthesis	Baud et al. (2007), Kuczynski et al. (2022)
6	*MYB96*	R2R3-type MYB Transcription factor	*FAE1, DGAT1, PDAT1*	Core seq CGAATAGTTACGGA	TAG assembly in seeds	Lee et al. (2018)
7	*bZIP67*	Basic leucine zipper transcription factor family (ABRE-binding factor)	*FAD3, CRC, SUS2*	G-box ACGT	Storage reserve accumulation during seed maturation	Mendes et al. (2013)
8	*L1L*	LEC1-LIKE(L1L) Nuclear transcription factor (NF-Y) family	*FAD3, SUS2, CRC, bZIP67, bZIP12*	CCAAT-box	Seed storage protein accumulation	Yamamoto et al. (2010)
9	*AGL15 AGAMOUS- LIKE 15*	A MADS (MIKC) domain transcriptional regulator	*LEC2, ABI3, and FUS3*	5′UTRC ArG motif of form C[A/T]8G or C[A/T]7 GG	Seed development and somatic embryogenesis	Zheng et al. (2009)
(B)	Negative TF’s					
1	*MYB89*	R2R3-MYB family TFs	*WRI1, L1L, BCCP1, SAD, ENO1, KAS1, KCSII*	Not defined	Inhibits seed oil accumulation	Li et al. (2017)
2	*BZIP10, BZIP25, BZIP53*	bZIP transcription factor family	*ABI3, CRU3, 2S2*	G-box element in MAT gene promoters	Key regulator of seed MAT gene transcription	Jain et al. (2017)
3	*BZIP52*	bZIP transcription factor family	*BCCP2, PKP-β1* *KASI, DGAT1*	WRI1, bZIP21 (bZIP domain through protein-protein interaction)	Represses fatty acid biosynthesis	Yang et al. (2023b)
4	*MYB76*	R2R3-MYB family TFs	*SUS4, SUC1, OLEOSIN3, AAD1, KCS17*	Not defined	Inhibits fatty acid biosynthesis in seeds	Li et al. (2017)
5	*TT8*	bHLH domain regulatory protein	*LEC1, LEC2, FUSCA3, CDS2, MOD1, FAE1, FAD2, FAD3*	Not defined	Inhibits seed fatty acid accumulation and reduced deposition of proteins in seeds	Chen et al. (2014)
(C)	Epigenetic Regulator					
1	*GCN5*	HAT GCN5, a GNAT-type HAT (GCN5-related N-acetyltransferase) HAT domain and a bromodomain	*FAD3, LACS2, LPP3 and PLAIIIβ*	Core promoter fragments (TATA box proximal region)	Decreased ALA increased the LA content	Wang et al. (2016)
2	*HSL1/VAL2*	B3 DNA binding domain, Plant homeodomain (PHD), zf- CW and EAR domain	*AGL15* *DOG1*	RY DNA motifs (CATGCA)	Represses the seed maturation	Chen et al. (2018b)
3	*HDA19 +HSL1*	B3 domain, zf-CW, and EAR	*DOG1* *LEC1* *OLE2*	RY-motifs (CATGCA)	Seed dormancy, seed germination and maturation	Zhou et al. (2013), Chen et al. (2020)
4	*PKL*	CHD3 chromatin remodelling, SNF2-relatedhelicase/ATPase domain, a DNA binding domain, and a PhD zinc finger	*ABI3 and ABI5*	Not defined	Limit the transcription programs	Perruc et al. (2007)
5	*CHR5*	CHD1-related gene	*FUS3 and ABI3*	5′UTR	Establishing the active state of embryo regulatory genes, reducing nucleosomal barrier to TSS.	Shen et al. (2015)

The AP2/ERF domain transcription factor Baby Boom (*BBM*), along with AINTEGUMENTA-LIKE (*AIL*), plays a critical role in regulating zygotic embryo development and the cellularization of the endosperm (Chen et al., 2022). CRISPR *bbm-cr* mutants showed their importance in embryo patterning (Chen et al., 2022).

LEAFY COTYLEDON1 (*LEC1*) acts as a molecular signal, transmitting cues from the endosperm to the embryo, thereby initiating various developmental programs essential for embryo maturation (Song et al., 2021). Mutations disrupting *LEC1* lead to reduced desiccation tolerance and lipid accumulation in soybean seeds (Pelletier et al., 2017; Jo et al., 2019). These mutations also lead to chromatin de-repression and the subsequent activation of *LAFL* genes, which are pivotal in initiating embryogenesis within the embryo of *Arabidopsis* seeds (Song et al., 2021). *LEC2*, activated by *LEC1*, plays a crucial role in signaling early embryogenesis, establishing an ideal cellular environment that supports both morphogenesis and subsequent seed maturation (Figures 2A, B). This gene is integral not only in the early stages of embryo formation but also during the later phases of seed development (Stone et al., 2001).

*LEC2*, along with *FUS3* and *ABI3*, operates as a central regulator of seed maturation, with each factor interdependently influencing the others. Notably, *LEC2* can activate both *LEC1* and *FUS3* to kickstart the maturation process (Stone et al., 2008; Liu B. et al., 2021). Moreover, *LEC2* can directly induce *AGL15*, a positive regulator of *LAFL*, and regulate the expression of gibberellic acid-related genes crucial for seed maturation (Stone et al., 2008) (Figure 2). *LEC2* also exerts control over *WRINKLED1 (WRI1)*, a master regulator of fatty acid synthesis, thereby playing a key role in fatty acid accumulation in oilseeds (Baud et al., 2007). Conversely, repressors such as *TCP4* and *MED15* act as repressors of *WRI1*, thereby inhibiting seed oil accumulation (Kong et al., 2020) (Figures 2A, B).

Seed maturation prepares the seed for germination, though this readiness is held in check until the seed encounters a suitable environment. ABI3, a seed-specific transcription factor, is instrumental in preparing seeds for dormancy, desiccation tolerance, and long-term viability (Delmas et al., 2013; Bedi et al., 2016; Tian et al., 2020). The intricate network of transcription factors that regulate seed embryogenesis, maturation (including lipid and protein accumulation), and dormancy represents a potential target for gene-editing technologies. Such interventions could enhance or biofortify oil content or yield cultivated plant varieties with improved tolerance to abiotic and biotic stresses.

### 4.2 Leafy Cotyledon1/Leafy Cotyledon2 (*LEC1/LEC2*)

*Leafy Cotyledon1* (*LEC1*), a subunit of the *NF-Y* transcription factor, plays a pivotal role in embryogenesis and seed maturation (Boulard et al., 2018). *LEC1* plays a critical role during seed maturation by coordinating the expression of various seed maturation genes. *LEC1* and its interactions with *AFL* transcription factors (*ABI3, FUS3, and LEC2*) form a regulatory network directing embryo development (Verma et al., 2022). *LEC1* expression in the endosperm can activate maturation genes even without embryo-expressed *LEC1*, reinforcing the endosperm’s role in seed maturation (Song et al., 2021) (Table 1; Figure 2B).

The *AFL* transcription factors play specific roles in activating genes responsible for seed storage protein synthesis, further reinforcing their importance in seed maturation (Yang T. et al., 2023). In *Arabidopsis*, *LEC1* mutant plants exhibit underdeveloped seeds characterized by short embryo axes, underdeveloped cotyledons with anthocyanin accumulation, and desiccation intolerance. Conversely, overexpression of *LEC1* leads to an upregulation of fatty acid biosynthetic genes, resulting in increased fatty acid levels. Additionally, *LEC1* enhances the expression of *FUS3, ABI3,* and *LEC2*, which regulate genes involved in photosynthesis and other developmental processes during early and late seed maturation (Jo et al., 2019).

*LEC1*’s role in the regulatory hierarchy of seed maturation is further emphasized by its interactions with other transcription factors. It interacts sequentially with different transcription factors, such as *LEC2 and ABI3,* through the B2 domain to activate specific seed development genes, although it does not interact with *FUS3,* which lacks the B2 domain (Boulard et al., 2018) (Figure 2A). The interaction between *LEC1* and *LEC2* is highly specific, with an amino acid residue (D86) being crucial for this interaction (Boulard et al., 2018). The translocation of the LEC2/LEC1/NF-YC2 complex from the cytoplasm to the nucleus is critical for activating *LEC2* and regulating downstream genes (Boulard et al., 2018). *LEC2*, another central embryonic regulator with a B3 domain, is vital for establishing the cellular environment necessary for embryo formation and later stages of seed development (Stone et al., 2001).

Although *LEC2* is primarily expressed during early embryogenesis, it remains essential for normal development throughout morphogenesis and maturation (Liu Q. et al., 2021). *LEC2* specifically binds to the conserved RY element (5′TAGAC-3′) in the promoter region of almost all seed-specific genes in plants. DNA microarray experiments during *Arabidopsis* seed development revealed that ectopic induction of *LEC2* in seedlings led to a rapid accumulation of RNAs encoding seed-specific proteins, such as 2S and 12S storage proteins, oleosin, and steroleosin, all typically expressed during seed maturation. These genes were significantly enriched with the RY motif within 1 kb upstream of the transcription start site, and DNA binding assays confirmed *LEC*2’s specific binding to this element (Braybrook et al., 2006).

Mutant lines lacking *LEC2* in *Arabidopsis* showed altered embryo morphology, changes in seed protein profiles, and desiccation intolerance (Stone et al., 2001) *LEC2* functions upstream in the regulatory hierarchy, upregulating *LEC1, FUS3*, and *ABI3* during embryo maturation (Table 1; Figures 2A, B). It also regulates the WRINKLED1 (*WRI1*) transcription factor, which controls TAG biosynthesis (Baud et al., 2007). Loss-of-function mutants of *LEC1, LEC2,* and *FUS3* exhibit defects in embryo formation or partial conversion of cotyledons into leaves. Additionally, *LEC2* is reported to trigger metabolic steps that increase TAG accumulation in vegetative tissues upon induction of senescence in *Arabidopsis* leaves (Kim et al., 2015).

### 4.3 *Wrinkled1* (*WRI1*)

*WRINKLED1* (*WRI1*) is an *APETALA2 (AP2)/EREBP* (Ethylene Response Element Binding Protein) transcription factor that plays a critical role in seed oil biosynthesis during seed maturation (Kong et al., 2020). Loss-of-function mutants show reduced seed weight (by approximately 25%–40%) and fatty acid content (by around 45%–55%) (Baud et al., 2007). Specifically, there was an increase in the levels of end products of fatty acid synthesis and desaturation reactions, such as C18:3 and C22:1, while the levels of C18:1, C18:2, and C20:1 were decreased (Baud et al., 2007). Earlier studies in 1998 showed that the *WRI1* mutation could reduce seed oil content by about 80%. Homozygous wrinkled mutants were found to have impaired ability to incorporate sucrose and glucose into triacylglycerol, though they incorporated pyruvate and acetate at an increased rate (Focks and Benning, 1998). Comparative genetic studies suggest that the expression of genes involved in late glycolysis and fatty acid synthesis is reduced in *WRI1* mutants (Ruuska et al., 2002).

Orthologous *WRI1* genes have been identified in various dicot and monocot plants, including *B. napus*, *C. sativa*, *G. max*, *Z. mays*, and *H. annuus*. The *WRI1*-binding cis-element, known as the AW-box (CnTnG7[CG]; where, ‘n’ represents any nucleotide), has been identified in the promoters of genes regulated by *WRI1* (Maeo et al., 2009).

A notable variant of *WRI1*, *AtWRI1W74R*, has been reported to enhance oil synthesis in *Arabidopsis* seeds. This variant demonstrated about a 60% increase in TAG content when transiently expressed in *N. benthamiana* (Qiao et al., 2022). The *W74R* variant exhibits a tenfold increase in binding affinity to AW-box sequences and is highly conserved across *WRI1* orthologs in diverse plant species. When this *W74R* mutant was introduced into other plants, such as *B. napus*, *C. sativa*, *Z. mays*, and *G. max*, consistently resulted in enhanced TAG accumulation upon transient expression in *Nicotiana benthamiana* (Qiao et al., 2022).

The structure and interaction of the WRI1 protein with cis-elements in gene promoters have been characterized in *Arabidopsis*, using the dsDNA of the AW-box isolated from the promoter of one of *WRI1’s* target genes (Table 1). The WRI1 protein structure comprises two similar AP2 domains (each consisting of two antiparallel β-sheets flanked by an α-helix) linked by a V-shaped arrangement of two α-helices (Qiao et al., 2022).

Another significant regulator of fatty acid biosynthesis in *Arabidopsis* is the TEOSINTE BRANCHED/CYCLOIDEA/PROLIFERATING CELL FACTOR (*TCP4*). *TCP4* acts as a repressor of *AtWRI1*, thereby reducing seed oil accumulation (Figures 2A, B). *Arabidopsis* mutants with reduced TCP4 activity accumulated more seed oil than the wild type, whereas *Arabidopsis* plants overexpressing *TCP4* (expressing rTCP4) showed reduced expression of *AtWRI1* target genes (Kong et al., 2020).

### 4.4 *Abscisic Acid Insensitive 3* (*ABI3*)

*Abscisic Acid Insensitive 3* (*ABI3*) is crucial for seed maturation, desiccation tolerance, entry into the quiescent stage, and seed longevity. *ABI3* mutants, exhibit defects in desiccation tolerance and storage product accumulation. Although loss-of-function mutants of *ABI3* do not show defects in embryo formation, the seeds remain green and fail to develop desiccation tolerance (Nambara et al., 1995; Delmas et al., 2013).

The expression of *ABI3*, regulated by hormone abscisic acid, is essential for de-greening of seeds in oilseed crops, which is necessary for seed maturation and seed oil quality (Delmas et al., 2013). Recent global mapping of *ABI3* DNA binding sites using DNA chip immunoprecipitation-tiling array assays has provided further insights (Mönke et al., 2012; Lim et al., 2013; Bedi et al., 2016). Transcriptome analysis comparing *abi3* three to five mutants with wild-type *Arabidopsis* seeds revealed that genes regulated by *FUS3* and *ABI3* often contain at least one RY-element CATGCA sequence (Table 1) (Wang and Perry, 2013; Tian et al., 2020). Genes with overlapping binding sites for both *ABI3* and *FUS3* are notably overrepresented in lipid biosynthesis, seed-oil biosynthesis (77.04%), and seed dormancy (Figures 2A, B). Approximately 43 genes are directly bound by both *FUS3* and *ABI3* and are also induced in response to these factors, predominantly those involved in lipid storage. Additionally, *FUS3* has been shown to induce the expression of miRNA156, which prevents premature maturation of *Arabidopsis* seeds (Wang and Perry, 2013; Tian et al., 2020).

*ABI3* directly activates or induces the expression of genes and transcription factors related to desiccation tolerance, such as *PLATZ1, PLATZ2,* and *AGAMOUS LIKE67 (AGL67)* (Tian et al., 2020). It also induces *LEA* genes like *LEA14* and genes involved in seed longevity, such as SEED IMBIBITION PROTEIN2 and raffinose synthase. Other *ABI3*-regulated genes include *NYC1* and *STAY-GREEN2* (*SGR2*), which are involved in chlorophyll breakdown (Tian et al., 2020). Both *ABI3* and *FUS3* directly induce *WRI1* (Wang and Perry, 2013; Tian et al., 2020). *ABI3* also directly induces the *FAD3* gene, which is involved in the synthesis of ALA in plants (Tian et al., 2020). *ABI3* contains B1 and B2 domains for interaction with bZIPs and LEC1 proteins, as well as a B3 DNA binding domain (Tian et al., 2020). Additionally, ABI3 induces other seed-specific factors, such as *CRUCIFERIN1*, *CRUCIFERIN3*, and various *LEA* genes, including *LEA76, LEA6, DEHYDRIN LEA,* and *LEA-LIKE* (Bedi et al., 2016).

### 4.5 Basic leucine zipper transcription factors (bZIPs)

The basic leucine zipper *(bZIP)* family of plant transcription factors is one of the largest and most diverse, playing crucial roles in regulating various synthesis pathways related to plant growth, development, and responses to biotic and abiotic stresses (Yu et al., 2020). In *Arabidopsis*, approximately *78 bZIP* transcription factors have been identified. These factors typically bind to palindromic or pseudo-palindromic hexamer sequences with a core motif of 5′-ACGT-3' (Dröge-Laser et al., 2018).

Recent research has highlighted the role of *bZIP52* in seed oil accumulation. Overexpression of *bZIP52* in *Arabidopsis* led to a reduction in seed oil content, while CRISPR/Cas9-mediated knockout of *bZIP52* resulted in increased seed oil accumulation (Yang Y. et al., 2023). This suggests that *bZIP52* functions as a repressor of fatty acid biosynthesis. Specifically, *bZIP52* interacts with the AP2 domain of *AtWRI1* (residues 58–240), preventing its binding to the AW-Box sequence in the promoters of fatty acid synthesis genes. This interaction indicates that the *AtWRI1–bZIP52* module is a key regulator of seed oil biosynthesis (Yang Y. et al., 2023).

Additionally, the regulation of seed maturation in *Arabidopsis* has been shown to involve the *bZIP53* transcription factor and its heterodimerizing partners, *bZIP10* and *bZIP25* (Jain et al., 2017) (Table 1) (Figures 2A, B). Another *bZIP67* TF was found to be induced by *LEC1* during embryogenesis along with the transcriptional complex comprising *LIL* and *NF-YC2* (Mu et al., 2008; Mendes et al., 2013; Tian et al., 2024). This complex activates *ALA* production by regulating the *FAD3* gene. The binding of *bZIP67* to cis-elements in the *FAD3* gene promoter enhances expression, but this binding and subsequent activation depends on the assembly of *LIL* and *NF-YC2* (Mendes et al., 2013; Tian et al., 2024). Null mutants of *FUS3* and *ABI3* showed no direct requirement for the enhanced activation of *FAD3* achieved with the *bZIP67-LIL-NF-YC2* complex (Mendes et al., 2013). However, *LEC1* triggers high levels of C18:3 during *Arabidopsis* seed maturation, which, in conjunction with the induction of *FUS3*, either alone or cooperatively with *LEC2*, leads to the activation of *bZIP67, LIL*, and *NF-YC2*. This forms a transcription complex that binds to G-boxes in the *FAD3* gene promoter (Mendes et al., 2013).

### 4.6 R2R3-MYB transcription factor family

In *Arabidopsis*, the *R2R3-MYB* transcription factor *MYB89* functions as a negative regulator of seed oil accumulation (Li et al., 2017). Predominantly expressed during seed development, *MYB89* plays a crucial role in seed maturation (Dubos et al., 2010). Mutations in *MYB89* lead to a significant increase in total fatty acid levels during seed maturation. This increase is associated with the upregulation of the conversion of acetyl-CoA to malonyl-CoA, which maintains the flux of fatty acids (Li et al., 2024). Additionally, the expression of the *BCCP1* and *BIO2* genes is also elevated. In *MYB89* mutants, the expression of *PLA2* and *ROD1* genes is also increased (Table 1) (Figures 2A, B). The *PLA2* gene hydrolyzes the sn-2 position of phospholipids, preferentially targeting linoleoyl acyl chains. The *ROD1* gene encodes Phosphatidylcholine-diacylglycerol choline phosphotransferase, which facilitates the transfer of C18:1 to phosphatidylcholine for desaturation and the reverse transfer of C18:2 and C18:3 in the TAG biosynthesis pathway (Li et al., 2017).

*MYB89* directly represses *WRI, ENO1, BCCP1, KASI, KCS11,* and *PLA2* *α* , and indirectly suppresses the master regulator *L1L* (Li et al., 2017). It also indirectly regulates other key genes in the oil biosynthetic pathway, including *CYFBP, FAB2, BIO2, HD-L, SAD, KCS17, FAE1, FAD2, FAD3,* and *ROD1* (Li et al., 2017). Conversely, the R2R3-type transcription factor *MYB96* acts as a positive regulator of TAG assembly in *Arabidopsis* seeds by regulating two rate-limiting enzymes: acyl-CoA: DGAT1 and phospholipid: diacylglycerol acyltransferase 1 (PDAT1) (Yang T. et al., 2023). *MYB96* is highly expressed in developing seeds, and its overexpression leads to increased fatty acid or TAG accumulation (Table 1). Quantitative real-time reverse transcription–PCR (RT-qPCR) analysis revealed that *DGAT1, PDAT1*, and *FAD3* are upregulated in *MYB96*-overexpressing seedlings and downregulated in *myb96*-mutant lines (Lee et al., 2018). Reduced expression of *DGAT1* and *PDAT1* is observed in the *myb96-2* mutant, which produces dry seeds. *MYB96* directly binds to the promoter of *ABI4*, which regulates *DGAT1* (Lee et al., 2018). Additionally, *MYB96* upregulates the *FAE1* gene in overexpressing seeds and downregulates it in *MYB96*-deficient seeds (Lee et al., 2018).

## 5 Epigenetic regulation of seed oil biosynthesis genes

Epigenetics regulates gene expression through heritable but reversible mechanisms, affecting both mitotic and meiotic cells (Ding et al., 2022). Key processes include DNA methylation, histone modification, and RNA-directed DNA methylation (RdDM), which can modify gene expression without altering DNA sequences (Kumar and Rani, 2023).

In oilseed crops, seed maturation and development are tightly regulated by epigenetic mechanisms, with chromatin modifiers repressing seed-specific genes. This epigenetic regulation of seed-specific and fatty acid biosynthesis genes is complex and critical for proper seed development, affecting oil seed content and quality (Zhang and Ogas, 2009). For instance, DNA methylation and histone modifications can change gene accessibility, impacting transcription and seed oil accumulation. This intricate interaction underscores epigenetics’ role in optimizing seed oil production and crop performance, although it’s molecular mechanism is yet to be fully understood.

### 5.1 Histone acetyltransferase general control non-repressed protein 5 (GCN5)

Histone acetyltransferase General Control Non-Repressed Protein 5 (GCN5) plays a crucial role in regulating the *FAD3* gene in *Arabidopsis.* The enzyme FAD3, a microsomal ω-3 fatty acid desaturase, is primarily responsible for synthesizing C18:3 in seeds (Browse et al., 1993). According to Wang et al. (2016), GCN5 influences *FAD3* post-transcriptionally through acetylation of histone H3 at lysines 9 and 14 in the upstream regulatory region. This acetylation is essential for the proper expression of *FAD3*. Mutations in GCN5 lead to a decreased ALA to LA ratio in *Arabidopsis* seeds, highlighting its role in fatty acid composition. The constitutive expression of the *FAD3* gene in *gcn5* mutant seeds restores the ALA/LA ratio to normal levels. Conversely, overexpression of GCN5 enhances the ALA/LA ratio, indicating that GCN5 positively regulates ALA synthesis (Wang et al., 2016).

Chromatin immunoprecipitation sequencing (ChIP-Seq) and ChIP-qPCR analyses have shown that in *gcn5* mutant seeds, there is a downregulation of several fatty acid biosynthesis-related genes, including *FAD3, LACS2, LPP3*, and *PLAIIIβ*. Within the FAD3 promoter, three specific regions have been identified as sites of acetylation by H3K9 and H3K14: approximately 200–400 bp upstream of the transcription start site (P1 region), 0–200 bp upstream (P2 region), and within the first exon (P3 region). These regions exhibit significantly reduced acetylation in the *gcn5* mutant compared to wild-type seeds. Overall, GCN5 is crucial for maintaining the ALA/LA ratio in *Arabidopsis* seeds by regulating the expression of *FAD3*.

### 5.2 High level expression of sugar inducible gene 2 (*HSI2*) or (*HSI2/VAL1*) and *HSL1* or (HSI2 LIKE1) (HSI2/VAL2)

*HSI2/VAL1* interacts with the *AGL15* gene through a specific DNA sequence and histone modifications, which are crucial for the shift from mature seed to germinating seedling stages (Figures 2A, B). This interaction silences the expression of *LAFL* genes, which are key regulators of seed maturation, by repressing *AGL15*—a positive regulator of these genes. This repression is further assisted by other silencing factors, such as MSI1, a polycomb DNA packaging protein (Chen N. et al., 2018). Recent studies have shown that Histone Deacetylase 19 (HDA19) can directly bind to the chromatin of seed maturation genes. HDA19 interacts with HSL1 to repress seed maturation during germination, and the homozygous double mutant of both genes is embryonically lethal (Zhou et al., 2013). Additionally, the *DOG1* (Delay of Germination 1) gene, a regulator of seed dormancy, is also controlled by *HSI2* and *HSL1*. These proteins bind to RY elements in the promoter region of DOG1, recruiting polycomb proteins CLF and LHP1. This recruitment leads to histone methylation (H3K27me3), which represses *DOG1* and thus breaks seed dormancy (Chen et al., 2020).

### 5.3 Pickle (PKL)

PICKLE (PKL) is a chromatin-remodeler protein that plays a crucial role in the regulation of the *ABI3* and *ABI5* genes, both of which are essential for conferring osmotolerance and resistance to germination under high-stress conditions. PKL is responsible for the epigenetic repression of these genes during germination, particularly in the presence of abscisic acid (ABA). In *pkl* mutants, *ABI3* and *ABI5* are less associated with repressed chromatin, leading to a failure in proper gene silencing (Figures 2A, B). PKL restricts the transcriptional activity of embryogenesis genes by modifying chromatin, thereby maintaining the appropriate expression levels required for seed germination under stress (Perruc et al., 2007).

### 5.4 Chromo domain Like-1(CHR1)

Chromo Domain-Like 1 (CHR1), also known as CHR5, is an epigenetic regulator that plays a contrasting role to PKL during embryogenesis. CHR1 activates the embryo development program by downregulating the expression of key embryogenesis genes such as *LEC1, ABI3,* and *FUS3*, while promoting the accumulation of storage seed proteins (SSPs). *CHR1* expression is prominent during late embryogenesis, where it antagonizes the function of *PKL* by reducing nucleosome occupancy near the transcription start sites (TSS) of *ABI3* and *FUS3* genes, thereby facilitating their activation (Shen et al., 2015).

These two proteins, PKL and CHR1, function antagonistically to regulate the balance between gene repression and activation during seed germination and embryogenesis, ensuring proper seed development.

## 6 Transcription factors in oilseed crops other than *Arabidopsis*

Transcription factors are essential in regulating gene expression during seed formation stages in oilseed crops, impacting fatty acid biosynthesis, elongation, desaturation, and export. In soybean (*G. max*), a key leguminous oilseed crop, these regulatory mechanisms are well-demonstrated. For instance, Chen L. et al. (2018) showed that *GmWRI1a*, a soybean *WRI1* ortholog, is highly expressed in maturing seeds and promotes fatty acid accumulation by binding to the AW-box sequence in fatty acid metabolism gene promoters. Additionally, transcription factors *LEC1* and *ABI3*, crucial in seed development, work within a network involving *AREB3* and *bZIP67* to regulate gene expression during seed maturation. Although *LEC1* and *ABI3* do not directly interact, *AREB3* and *bZIP67* form heterodimers that link with the LEC1-NF-YC dimer, merging LEC1 and ABI3 into a functional complex, which finely tunes seed maturation gene expression (Jo et al., 2019).

Studies show that overexpression of Dof-type transcription factors *GmDOF4* and *GmDOF11* in soybean seeds increases lipid content by activating key metabolic genes like acetyl-CoA carboxylase and long-chain acyl-CoA synthetase. These factors also repress protein synthesis by binding to the *CRA1* promoter (Wang et al., 2007). GmDOF4’s ortholog, GmDOF4.2, is linked to drought tolerance in *N. tabacum* (Zhai et al., 2023).

In sesame, another oilseed crop often grown in drought-prone areas, the genome has 132 *AP2/ERF* genes. Dossa et al. (2016) reported that 23 *DREB* genes are upregulated in response to drought, indicating a role in stress tolerance. In oil biosynthesis, the *DREB*-type transcription factor *GmDREBL* activates the *WRINKLED* promoter, along with downstream oil biosynthesis genes. *GmDREBL* expression is regulated by *GmABI3* and *GmABI5*, suggesting that gene editing of *GmDREBL* could enhance oil content and improve stress resistance in oilseed crops (Zhang et al., 2016).

## 7 Genome editing techniques for precise genetic modifications

Genome editing (GE) is a precise technique employed to alter the genomes of various organisms, including yeast, bacteria, plants, and animals. Among the available tools for this purpose—such as zinc-finger nucleases (ZFNs) and transcription activator-like effector nucleases (TALENs)—CRISPR/Cas9 has emerged as a particularly effective and economical method for genetic modification, significantly enhancing crop yields (Negi et al., 2022; Cardi et al., 2023; Gawande et al., 2024). Prior to CRISPR/Cas9, methods like ZFNs were both costly and time-consuming (Cardi et al., 2023). Since its initial adaptation from bacterial immune systems for targeted DNA cleavage (Jinek et al., 2012), CRISPR/Cas9 has become central to genome engineering. Unlike meganucleases, ZFNs, and TALENs, which recognize target sequences through protein-DNA interactions, the CRISPR/Cas system employs Watson–Crick base pairing for DNA targeting. This system uses the Cas9 endonuclease in conjunction with a single-guide RNA (sgRNA) that includes a 20-base pair complementary sequence, guiding precise genome modifications through repair processes like nonhomologous end-joining (NHEJ) or homology-directed repair (HDR) (Jinek et al., 2012; Makarova et al., 2015; Cardi et al., 2023; Pan and Qi, 2023).

The CRISPR toolbox has since expanded to include various Cas nucleases with unique features and PAM recognition sites, which are employed for plant genome editing (Pramanik et al., 2021; Gawande et al., 2024). Cas9 from *Streptococcus pyogenes* (SpCas9) remains the most widely used due to its well-understood mechanism, which involves the Cas9-sgRNA complex searching for target DNA sequences with the protospacer adjacent motif (PAM). This complex binds to the target DNA, inducing a double-strand break (DSB) facilitated by the RuvC and HNH nuclease domains. Additionally, the development of Cas variants—such as nickase (nCas9) and dead Cas (dCas9)—has enabled the creation of CRISPR tools with diverse editing capabilities, including gene knockout, base editing, and epigenetic modifications (Gao, 2021; Pramanik et al., 2021; Wang and Doudna, 2023; Gawande et al., 2024; Shelake et al., 2024). Recently, a transposon-associated TnpB protein has been successfully employed as an alternative to Cas9 for genome editing in plants (Karmakar et al., 2024). Novel tools like CRISPR-TSKO also offer tissue-specific genome editing, allowing for spatially and temporally controlled genetic alterations (Decaestecker et al., 2019).

### 7.1 Current state of oilseed crop via CRISPR/Cas systems

Genome editing techniques have emerged as a superior method for enhancing oil quality in oil-producing crops, surpassing conventional breeding and genetic engineering approaches. Traditional breeding has long served as the foundation for crop improvement, focusing on selecting traits like yield, pest resistance, and adaptability. However, while breeding has achieved significant successes, it has limitations in precision and speed, especially with complex traits like oil composition and stress resilience. Recent advancements in transcriptional engineering and genome editing, such as CRISPR/Cas9, complement breeding by allowing specific, targeted modifications at the genetic level (Cardi et al., 2023; Gawande et al., 2024). These tools enable breeders to precisely regulate genes involved in traits like fatty acid biosynthesis and stress responses, accelerating the development of high-performing, resilient oilseed varieties. By integrating these methods, breeders can enhance traditional crop lines with desirable genetic traits more efficiently, meeting the increasing demand for improved nutritional profiles, higher yields, and robust environmental tolerance.

Traditional methods involving transgenics face limitations such as unintended genetic changes, reduced efficacy, lengthy processes, and the challenges of transgene insertion (Wang and Doudna, 2023). In contrast, the successful application of CRISPR/Cas9 in oil-producing crops has been well-documented (Table 2). Techniques like transcriptome analysis, map-based cloning, gene silencing, and pathway analysis have been instrumental in functionally validating target genes. In polyploid oilseed crops such as rapeseed, canola, camelina, peanut, and cottonseed, CRISPR technology enables the simultaneous editing of multiple genes (Yuan et al., 2019; Zhang et al., 2019; 2023; Chen et al., 2021; Neelakandan et al., 2022). This approach also facilitates the generation of stable knock-out mutants that can be backcrossed to develop transgene-free oilseed crops, significantly accelerating genetic improvements in these plants (Table 2).

**TABLE 2:** Genome editing in oil-producing crops.

S. No	CRISPR edited crops	Used editing technique	Genes	Transformation technique	Feature	References
1	Rapeseed	CRISPR/Cas9	*BnFAD2*	AMT	Increased the oleic acid, fatty acid content	Okuzaki et al. (2018), Huang et al. (2020)
2	Rapeseed	CRISPR/Cas9	*BnGTR2*	AMT	High oil content	Tan et al. (2022)
3	Rapeseed	CRISPR/Cas9	*BnLPAT2/5*	AMT	Seeds showed enlarged oil bodies, disrupted distribution of protein bodies, and increased accumulation of starch in mature seeds	Zhang et al. (2019)
4	Rapeseed	CRISPR/Cas9	*BnTT2*	AMT	Improved fatty acid composition	Xie et al. (2020)
5	Rapeseed	CRISPR/Cas9	*BnaFAE1*	AMT	Low erucic acid content and increased oleic acid	Liu et al. (2022b)
6	Rapeseed	CRISPR/Cas9	*BnFAD2,* and *BnFAE1*	AMT	Increased oleic acid	Shi et al. (2022)
7	Rapeseed	CRISPR/Cas9	*SFAR*	AMT	Seed oil content increased	Karunarathna et al. (2020)
8	Soybean	CRISPR/Cas9	*FAD2-2*	AMT	Increase of oleic acid content and decrease of linoleic acid	(Al Amin et al., 2019; Do et al., 2019
9	Soybean	CRISPR/Cas9	GmFAD2-1A and GmFAD2-2A	AMT	Increase of oleic acid content	Wu et al. (2020)
10	Soybean	CRISPR/Cas9	*GmFATB1*	AMT	Significant reduction of amount of saturated fatty acids in seeds	Ma et al. (2021)
11	Soybean	CRISPR/Cas9	*GmFAD2-1B* *GmFAD2-1A, GmFAD2-2B, GmFAD2-2C,* and *GmFAD2-2D*	AMT	Increased oleic acid content and decreased linoleic acid	Zhang et al. (2023)
12	Cotton	CRISPR/Cas9	*GhFAD2*	AMT	High-oleic-acid portion	Chen et al. (2021)
13	Cotton	CRISPR/Cas9	GhBLH1, *GhFAD7A-1-OE*	AMT	Enhances linolenic acid accumulation	Jia et al. (2024b)
14	Peanut	CRISPR/Cas9	*AhFAD2*	AMT	Increased oleic acid content	Yuan et al. (2019), Neelakandan et al. (2022)
15	Peanut	TALEN	*AhFAD2*	AMT	Mutagenesis of fatty acid desaturase 2	Wen et al. (2018)
16	Peanut	CRISPR/Cas9	*AhFatB*	AMT	High oleic acid and low palmitic acid content	Tang et al. (2022)
17	Peanut	CRISPR/Cas9	*AhKCS1* and *AhKCS28*		Reduced VLCFAs levels	Huai et al. (2024)
18	False flax	CRISPR/Cas9	*CsFAD2*	AMT	High oleic acid content	Jiang et al. (2017), Morineau et al. (2017)
19	False flax	CRISPR/Cas9	*CsDGAT1/CsPDAT1*	AMT	Reduced oil content	Aznar-Moreno and Durrett (2017)
20	False flax	CRISPR/Cas9	*LEC1, LEC2, FUS3, WR1*	AMT	Increased fatty acid synthesis rates, TFA accumulation, and TAG yield	Cai et al. (2024)
21	Field cress	CRISPR/Cas9	*FAE1, FAD2, and ROD1*	PEG-mediated protoplast transfection	Increased oleic acid	Sandgrind et al. (2023)
22	Rice	CRISPR/Cas9	*OsFAD2-1*	AMT	High oleic/low linoleic	Abe et al. (2018)
23	Palm	CRISPR/Cas9	*EgFAD2* and *EgPAT*	Protoplast, biolistic, and AMT	High oleic acid content	Bahariah et al. (2021), 2023
24	Pennycress	CRISPR/Cas9	*FAD2, FAE1, ROD1*	AMT	Increase oleic acid content	Jarvis et al. (2021)

Note: AMT (Agrobacterium-mediated transformation).

#### 7.1.1 Peanut (*Arachis hypogaea* L.)

Peanut, an allotetraploid (AABB) crop with 2n = 4x = 40 chromosomes, is an important economic resource due to its high-quality oil yield (Husted, 1936; Bertioli et al., 2019; Leal-Bertioli et al., 2021). According to the United States Department of Agriculture (USDA), global peanut production in 2024 reached 51.31 million metric tons (https://ipad.fas.usda.gov). The quality of peanut oil is determined by the ratio of saturated fatty acids (SFAs) to making up the remaining 80% (Wang et al., 2015; Shasidhar et al., 2017; Tang et al., 2022).

**FIGURE 3:**
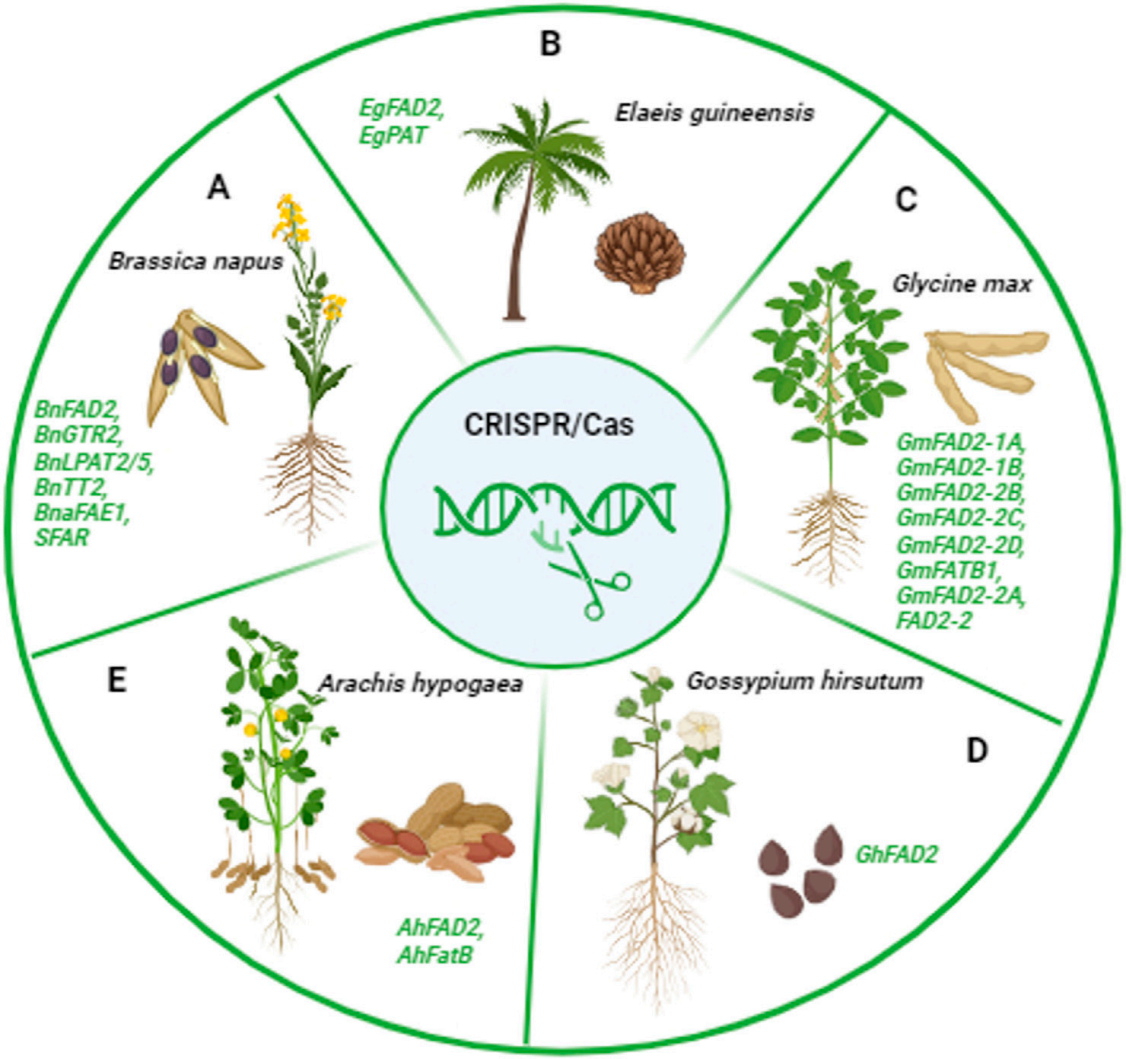
Schematic representation of the different CRISPR-edited oilseed crops. **(A)** *BnFAD2, BnGTR2, BnLPAT2/5, BnTT2, BnaFAE1, SFAR* genes responsible for oil quality improvement by CRISPR in *Brassica napus*; **(B)** *EgFAD2, EgPAT* genes responsible for oil quality improvement by CRISPR in *Elaeis guineensis*; **(C)** *GmFAD2-1A, GmFAD2-1B, GmFAD2-2B, GmFAD2-2C, GmFAD2-2D, GmFATB1, GmFAD2-2A, FAD2-2* genes responsible for oil quality improvement by CRISPR in *Glycine max*; **(D)** *GhFAD2* gene responsible for oil quality improvement by CRISPR in *Gossypium hirsutum;* **(E)** *AhFAD2, AhFATB* genes responsible for oil quality improvement by CRISPR in *Arachis hypogaea.* The figure is created with BioRender.com.

In 2018, Wen et al. successfully used transcription activator-like effector nucleases (TALENs) to modify the *FAD2* gene in peanuts, resulting in mutant varieties with significantly higher oleic acid content (90.45%) in their oil. This modification also led to a decrease in linoleic acid levels by 3%–19% in *fad2* T2 transgenic seeds compared to the wild type, marking the first successful instance of genome editing in peanuts and showcasing the potential of TALEN-induced mutagenesis in peanut breeding (Table 2) (Wen et al., 2018). Additionally, 18 *AhFatB* genes identified in the *A. hypogaea* genome, which are crucial for glycerolipid content and play essential roles in seed and flower development, were studied. The CRISPR/Cas9 system revealed mutations at *Arahy.4E7QKU*, resulting in low palmitic acid and high oleic acid phenotypes (Tang et al., 2022) (Table 2). Similar results were observed when CRISPR/Cas9 identified three mutations—G448A in *ahFAD2A,* 441_442insA, and G451T in *ahFAD2B*—with G451T being a novel mutation and G448A and 441_442insA previously identified in high oleate variants (Yuan et al., 2019) (Table 2) (Figure 3).

Further advancements in peanut genome editing were demonstrated through base editing using CRISPR/nCas9 technology (Neelakandan et al., 2022). Researchers constructed two expression vectors, pDW3873 and pDW3876, by fusing Cas9 to different deaminases to induce point mutations in the promoter and coding sequences of the *AhFAD2* genes. These constructs were tested for their ability to replace cytosine with thymine or other bases in the targeted editing window. The pDW3873 vector showed higher efficiency, indicating its potential as a superior base editor in peanuts. This technique could significantly shorten the time required to introgress beneficial mutations into elite peanut varieties, offering substantial benefits to breeders, farmers, and consumers alike.

In addition, a recent study by Huai et al. (2024) investigated reducing very long-chain fatty acids (VLCFAs) in peanut kernels using CRISPR-Cas9 technology. Natural high levels of VLCFAs in peanuts are detrimental to human health, being linked to cardiovascular diseases. Previous studies identified *AhKCS1* and *AhKCS28* as putative regulators of VLCFA content in peanut kernels. The biosynthesis of VLCFAs in plants is regulated by β-ketoacyl-CoA synthase (KCS) (Wang et al., 2017). In a prior study, 30 *AhKCS* genes were identified in peanut genomes, with *AhKCS1* and *AhKCS28* found to be key regulators of VLCFA content. The VLCFA content in available peanut germplasm ranges from 4.3% to 9.8%, with no sequence variation observed within or surrounding the *AhKCS1* and *AhKCS28* genes, suggesting that gene editing is the only viable method for further reduction. Consequently, *AhKCS1* and *AhKCS28* were genetically disrupted using the CRISPR/Cas9 system, producing novel peanut mutants with significantly reduced VLCFA levels. The results demonstrated up to a 100% reduction in VLCFA content in the double mutants, underscoring the efficacy of CRISPR-Cas9 in modifying peanut fatty acid profiles and highlighting its potential in enhancing crop genetics for global food security and human health (Huai et al., 2024).

#### 7.1.2 Soybean (*Glycine max*)

Soybean, a polyploid (2n = 4x = 40) legume and essential oil crop (Ren et al., 2021; Yuan and Song, 2023), is cultivated on about 120 million hectares worldwide (Berbenets et al., 2020), with global production nearing 352 million tons (Voora et al., 2020; Sokolovskaya and Valevskaya, 2023). Enhancing oleic acid content and improving the oleic-to-linoleic (O/L) ratio in soybean is a major breading goal (Monteros et al., 2008). Using CRISPR/Cas9, researchers have edited five *FAD2* genes (*GmFAD2-1B, GmFAD2-1A, GmFAD2-2B, GmFAD2-2C,* and *GmFAD2-2D)* in soybean varieties (JN38, T6098, and T7010), resulting in higher oleic acid levels (54.07%) and lower linoleic acid levels (26.17%) (Zhang et al., 2023) (Table 2), showing that targeting multiple *FAD2-2* genes can enhance soybean oil’s nutritional quality. Al Amin et al. (2019) also used CRISPR-Cas9 to modify the *FAD2-2* gene, achieving 65.58% oleic acid and 16.08% linoleic acid (Table 2).

Do et al. (2019) used CRISPR/Cas9 with two gRNAs to target separate exons of *GmFAD2-1A* and *GmFAD2-1B*, achieving CRISPR-edited DNA in 77.8% of T0 plants, with edits inherited in T1 progeny. This resulted in a 1.3%–2.7% decrease in linoleic acid and over 80% oleic acid content. Wu et al. (2020) observed similar results in T2 and T3 generations. Additionally, Ma et al. (2021) used CRISPR/Cas9 to create mutants of the *FATB* genes, *GmFATB1a* and *GmFATB1b*, which resulted in reduced stearic and palmitic acids and a 1.3%–3.6% increase in linoleic acid (Table 2) (Figure 3).

#### 7.1.3 Rapeseed (*Brassica napus*)

Rapeseed (*B. napus*, allotetraploid, 2n = 4x = 38), is a major global source of edible oils and is valued for its nutritional profile and biodiesel potential (Dupont et al., 1989; Ohlrogge, 1994; Thelen and Ohlrogge, 2002; Sun et al., 2017; Liu Y. et al., 2022; Shi et al., 2022). According to USDA data, global rapeseed production in 2023/2024 reached 89.33 million metric tons (https://ipad.fas.usda.gov).

CRISPR/Cas9-mediated editing of the *BnFAD2* and *BnFAE1* genes has enhanced the nutritional quality of the rapeseed cultivar CY2, which contains roughly 50% oil content and 40% erucic acid (Shi et al., 2022). These edits increased oleic acid content by 70%–80% and reduced erucic acid levels in lines with mutations at all target sites (Shi et al., 2022) (Table 2). Similarly, Okuzaki et al. (2018) reported that CRISPR/Cas9-modified *FAD2_Aa* mutants achieved oleic acid concentrations of 73%–80% and lower linoleic acid levels of 16%–9%, with corroborating results from Huang et al. (2020) (Table 2).

Targeted knockout of *BnaFAE1* has significantly improved the nutritional quality of *B. napus* seed oil, especially in *BnaA08.fae1* and *BnaC03.fae1* double mutants, which resulted in a ∼66% increase in oleic acid content (Liu Y. et al., 2022) (Table 2). Xie et al. (2020) utilized CRISPR/Cas9 to target Transparent Testa (*TT*) genes in *B. napus*, leading to reduced flavonoid levels, increased oil content, and enhanced fatty acid composition with higher linoleic (C18:2) and linolenic acids (C18:3) (Table 2). CRISPR-derived *Bna.gtr2* mutants, including the double mutant *BnaC03.gtr2* and its three paralogs, positively regulated seed glucosinolate accumulation, resulting in smaller seeds with higher oil content (Tan et al., 2022) (Table 2). Zhang et al. (2019) used CRISPR/Cas9 to target Lysophosphatidic Acid Acyltransferase (*LPTA*) genes, *BnLPAT2* and *BnLPAT5*, leading to increased oil body size and decreased oil content, highlighting their roles in oil biosynthesis (Table 2) (Figure 3).

#### 7.1.4 Cotton (*Gossypium hirsutum*)

Cotton (allotetraploid, 2n = 4x = 52) is the world’s fifth-largest source of vegetable oil and serves as a significant cash crop for natural textiles (Chen et al., 2021). According to USDA, global cotton production for 2023/2024 is projected to reach 113.66 million bales (https://fas.usda.gov/data/production/commodity/2631000). The oil extracted from cottonseed contains 26% palmitic acid, 15% oleic acid, and 58% linoleic acid (Liu et al., 2002; Chen et al., 2021). In a recent study, Chen et al. (2021) used CRISPR/Cas9 to delete the *GhFAD2* gene, resulting in mutants with elevated oleic acid content, marking the first successful production of high-oleic cotton using this technology (Chen et al., 2021) (Table 2) (Figure 3). Additionally, another recent study Jia et al. (2024) identified the BEL1-LIKE HOMEODOMAIN (*BLH*) gene *GhBLH1*, which positively regulates cotton fiber cell elongation. Their research revealed that *GhBLH1* enhances linolenic acid accumulation by activating the *GhFAD7A-1* gene, also leading to longer fiber cells. This finding highlights the role of long-chain and very-long-chain fatty acids in fiber cell elongation and underscores the importance of linolenic acid in cotton fiber growth. The molecular mechanism uncovered in this study involves the binding of the POX domain of GhBLH1 to the TGGA cis-element in the GhFAD7A-1 promoter, leading to increased transcription and subsequently, longer fiber cells. Conversely, knockout of *GhFAD7A-1* using CRISPR/Cas9 results in shorter fibers, while its overexpression promotes fiber elongation. These studies exemplify how targeted genetic modifications can influence key traits in crops, providing insights into the genetic and molecular controls of oil and fiber production in cotton.

#### 7.1.5 *Camelina sativa*

*Camelina sativa*, an allohexaploid oilseed from the *Brassicaceae* family, is increasingly valued for its diverse applications (Nguyen et al., 2013; Morineau et al., 2017; Lee et al., 2021; Lin et al., 2022). However, its high linolenic acid content (30%–40%) limits its use in biofuels, lubricants, and food products (Nguyen et al., 2013; Iskandarov et al., 2014; Morineau et al., 2017; Lee et al., 2021).

CRISPR/Cas9 gene editing has been used to modify *FAD2* genes in *Camelina*, increasing oleic acid levels from 16% to over 50% while reducing linoleic acid (16% to <4%) and linolenic acid (35% to <10%) (Table 2) (Morineau et al., 2017). Similar results were achieved with a CRISPR-Cas9 triple *CsFAD2* knockout, which showed an 80% increase in oleic acid compared to the wild type (Lee et al., 2021) (Table 2). Additionally, modifying triacylglycerol synthesis genes *CsDGAT1* or *CsPDAT1* in *Camelina* seeds has led to changes in oil content and fatty acid composition (Aznar-Moreno and Durrett, 2017) (Table 2).

In a recent study, Cai et al. (2024) utilized CRISPR/Cas9 to disrupt the Transparent Testa 8 (*TT8*) transcription factor genes in *Camelina*, resulting in a yellow seed phenotype due to reduced flavonoid accumulation (up to 44%) and a disrupted seed coat mucilage layer. Their study identified and edited three *TT8* genes, one from each *Camelina* subgenome, creating independent CsTT8 lines with frameshift mutations. This genetic modification led to significant upregulation of lipid-related transcription factors (*LEC1, LEC2, FUS3, and WRI1*) and their targets, enhancing fatty acid synthesis. Consequently, total fatty acid (TFA) content increased from 32.4% to as high as 38.0% of seed weight, and TAG yield rose by over 21%, with no significant changes in starch or protein levels compared to the parental line (Table 2).

These findings underscore the potential of CRISPR/Cas9 to create high-oil *Camelina* lines, contributing to future sustainable biofuel production and the development of high-yield lipid-derived bioproducts.

#### 7.1.6 Pennycress (*Thlaspi arvense L.*)

Pennycress is a homozygous diploid species (2n = 2x = 14) (Navarrete et al., 2022; Nunn et al., 2022) that is native to Eurasia and has since become widely distributed globally (Best, 1978; Sharma et al., 2007; Jarvis et al., 2021). In their research, Jarvis et al. (2021) utilized CRISPR/Cas9 technology to enhance the oleic acid content in pennycress seed oil by targeting the fatty acid desaturase 2 (*FAD2*) and reduced oleate desaturation 1 (*ROD*1) genes. The resulting double knockdown mutants demonstrated a significant increase in oleic acid production, highlighting a promising strategy for improving commercial yields.

#### 7.1.7 Palm (*Elaeis guineensis Jacq.*)

*Elaeis guineensis* is a diploid species with 16 chromosomes (Low et al., 2024). According to the USDA, global palm oil production for the 2023/2024 season is projected to reach 77.28 million metric tons, with Indonesia being the world’s largest producer, accounting for 57% of this output (https://fas.usda.gov/data/production/commodity/4243000). In Malaysia, the oil palm is a crucial commodity crop that contributes to 35% of global oil and fats production (Bahariah et al., 2021; Parveez et al., 2021; Bahariah et al., 2023; Norfaezah et al., 2024; Ramesh et al., 2024). In their study, Bahariah et al. (2023) employed the CRISPR/Cas9 technology to create knockout mutants with enhanced oleic acid content by targeting the *EgFAD2* and palmitoyl-acyl carrier protein thioesterase (EgPAT) genes in oil palm. The Cas9/sgRNA genome-editing approach effectively knocked out both genes, resulting in the generation of single- and double-knockout mutants (Bahariah et al., 2021; Bahariah et al., 2023) (Table 2) (Figure 3).

## 8 Perspectives

Transcriptional engineering represents a revolutionary approach to enhancing the value of oilseed crops, focusing on both improving oil quality and increasing yield. Advances in gene editing technologies, particularly CRISPR/Cas9, along with synthetic biology tools, have enabled precise modifications of the transcriptional networks governing oil biosynthesis and storage. By combining transcriptional engineering with precision agriculture practices, it is possible to develop crop varieties specifically tailored to distinct environmental conditions and farming practices, thereby enhancing resource use efficiency, reducing environmental impact, and improving crop resilience. Furthermore, developing oilseed crops that can withstand extreme weather conditions and fluctuating climates is critical due to the detrimental impact of climate change on crop yield. Moreover, engineering crops to enhance their nutritional profiles, such as increasing the content of essential fatty acids and other beneficial compounds, can provide significant health benefits and help address nutritional deficiencies in various populations.

The successful application of CRISPR/Cas technology on rapeseed, soybean, cotton, camelina, pennycress, and palm, has demonstrated remarkable potential for enhancing oil quality and nutritional value. By targeting specific genes involved in fatty acid biosynthesis and composition, researchers have been able to develop crop variants with improved oil profiles. By precisely modulating the expression of genes involved in stress tolerance mechanisms, such as heat shock proteins (HSPs), researchers can develop crop varieties with improved resilience to heat stress and other abiotic stresses. Future research in this field should prioritize the simultaneous improvement of multiple traits, including yield, oil quality, and stress resistance. This holistic approach necessitates the use of sophisticated gene stacking techniques and advanced regulatory network analyses to achieve comprehensive enhancements. Additionally, synthetic biology can introduce new biosynthetic pathways into oilseed crops, allowing for the production of novel oils and value-added compounds. This innovation could create new markets and applications for oilseed-derived products, expanding the economic potential of these crops. Moreover, the development of transgene-free oilseed crops through CRISPR-mediated genome editing offers a promising avenue for ensuring the safety and acceptance of genetically modified organisms in agriculture. Despite the progress made in utilizing CRISPR/Cas9 for crop improvement, challenges remain, particularly in oilseed crops with complex genomes and resistance to regeneration like cotton and peanut, which pose technical barriers to efficient genome editing.

Comparative evaluations reveal that while some crops respond robustly to transcriptional interventions, others require additional optimization to overcome genetic complexity and regeneration difficulties. For instance, in *Brassica napus* (rapeseed), CRISPR-based transcriptional modifications have notably increased oleic acid content and decreased undesirable fatty acids like erucic acid. This progress addresses both nutritional quality and regulatory standards, making rapeseed oil more appealing for food and industrial applications (Shi et al., 2022). In contrast, *Gossypium hirsutum* (cotton) presents greater complexity, as its polyploid genome has proven more challenging for stable transcriptional edits. However, recent breakthroughs have shown potential in enhancing oleic acid levels and fiber quality simultaneously, suggesting that multiplexed editing strategies could unlock further gains in cotton (Chen et al., 2021).

*Glycine max* (soybean) has also benefited from transcriptional engineering with relatively straightforward genome editing, leading to increased oleic acid and reduced linoleic acid content, improving oil stability and shelf life (Do et al., 2019). Soybean’s amenability to editing has made it a model for other legumes and oilseed crops, showcasing how regulatory transcription factors, when edited, can achieve specific oil profile enhancements. By contrast, *Arachis hypogaea* (peanut) has posed unique challenges due to its allotetraploid genome structure, which complicates the precise targeting of transcriptional elements. Despite these hurdles, targeted editing of fatty acid desaturase genes in peanuts has produced high-oleic varieties with better oil quality, although achieving stable, heritable modifications remains an ongoing challenge (Tang et al., 2022).

In *Camelina sativa*, a relatively new candidate for industrial oils and biofuels, transcriptional modulation has led to significant increases in oleic acid and reductions in polyunsaturated fatty acids, making it suitable for biofuel production (Morineau et al., 2017). *Camelina’s* simpler genome structure and compatibility with genetic modification approaches position it as a promising oilseed for future applications. *Thlaspi arvense* (pennycress), while less studied, has demonstrated potential as a biofuel feedstock through CRISPR-mediated modulation of fatty acid pathways, increasing oleic acid while reducing undesirable fatty acids, though further research is needed to fully harness its agronomic potential (Jarvis et al., 2021).

These comparative insights emphasize that while transcriptional technologies offer universal benefits for oilseed crop improvement, species-specific challenges, such as polyploidy and regeneration efficiency, require tailored approaches (Pramanik et al., 2021). Moving forward, a balanced approach that combines genome editing with advances in transcriptomics and synthetic biology will allow for more comprehensive enhancements across diverse oilseed crops. This trajectory not only meets the growing demand for nutritionally rich oils but also ensures the sustainability and economic viability of oilseed production under changing climatic conditions.

However, ongoing research efforts aim to overcome these challenges and unlock the full potential of genome editing technologies for enhancing the resilience and productivity of oilseed crops in the face of climate change.

Furthermore, as ‘omics’ technologies unravel the intricacies of lipid metabolism in oilseeds, a deeper understanding of regulatory networks emerges. Integration of genomic, transcriptomic, proteomic, and metabolomic data offers invaluable insights into enhancing seed oil quality, pivotal for future crop enhancement endeavours. Harnessing transcriptional and epigenetic regulation mechanisms presents further avenues for crop improvement through targeted breeding and genetic engineering.

## 9 Conclusion

In conclusion, by embracing cutting-edge biotechnologies and addressing the associated challenges, we can pave the way for a sustainable and prosperous future for oil crop improvement. Ongoing research and innovations in transcriptional engineering will contribute to the next green revolution, ensuring food security and environmental sustainability for future generations. This comprehensive approach will not only meet the escalating global demand for vegetable oils but also ensure the economic viability and ecological sustainability of oilseed crop production.
